# A Mild Synthesis of New Aryl Vinyl Ethers and Diethyl 1-[(Alkyl)(cyano)methyl]vinylphosphonates via the Substitution of a 2,3-Difunctional Allyl Bromide

**DOI:** 10.1155/2014/260325

**Published:** 2014-02-12

**Authors:** Asma Fray, Jihène Ben Kraïem, Aïcha Arfaoui, Hassen Amri

**Affiliations:** ^1^Selective Organic Synthesis & Biological Activity, Faculty of Science, El Manar University, 2092 Tunis, Tunisia; ^2^Laboratoire Méthodes et Techniques d'Analyse (LMTA), Institut National de Recherche et d'Analyse Physico-Chimique (INRAP) Biotechnopôle Sidi-Thabet, 2020 Tunis, Tunisia

## Abstract

A novel class of aryl vinyl ethers **3** and diethyl 3-cyano-3-alkylprop-1-en-2-ylphosphonates **4** has been prepared, respectively, from coupling reaction of diethyl 1-(bromomethyl)-2-cyanovinylphosphonate **2** with phenols and Gilman reagents.

## 1. Introduction

**Scheme 1:**
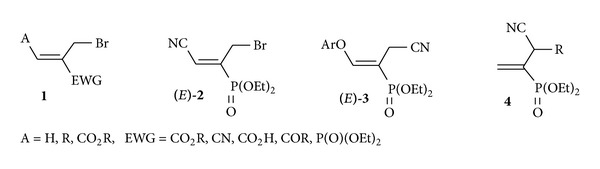
Target functional vinylphosphonates **3** and **4** obtained from allyl bromide **2**.

The development of new strategies for the synthesis of organic compounds containing an *α*-bromomethyl group as in product **1** continues to pose a challenge to organic chemists. It has resulted in a wide variety of applications in the preparation of several natural [1–4] as well as biologically active compounds [5–8]. Moreover, the utility of these versatile allyl brominated intermediates **1** [9–18] comes from their reaction with different nucleophilic species and additionally their ability to act as excellent Michael acceptors. Because of its importance, it is still of interest to develop novel approaches for the efficient generation of functionalized allyl bromide **1**. In this regard, we have previously described a simple and stereoselective synthesis of diethyl (*E*)-1-(bromomethyl)-2-cyanovinylphosphonate **2** [19], an activated alkene bearing three functional groups. We have also demonstrated that the latter can be used as an efficient electrophilic synthon for the synthesis of a new family of allylamines [19], enamines [20], and vinyl ether [20]. Prompted by the versatility of allyl bromide **2**, we suggested that its reaction with a variety of phenols and Gilman reagents would be a very convenient way to prepare new substituted aryl vinyl ethers **3** and diethyl 1-[(alkyl)(cyano)methyl]vinylphosphonates **4** (Scheme 1).

## 2. Results and Discussion

**Scheme 2:**
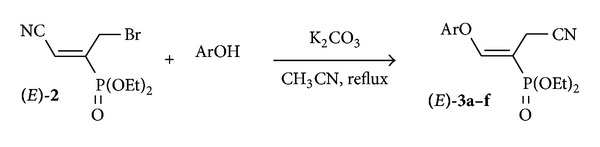
Basic *O*-vinylation of phenols with allyl bromide **2**.

Functional aryl vinyl ethers have been shown to be promising building blocks in organic synthesis [21–23] as well as important intermediates for the synthesis of a range of products [24–33], new polymeric materials [34–36], and biologically active molecules [37]. Thus, the preparation of these kinds of molecules has always attracted the attention of organic chemists and a growing effort has been directed toward the development of new vinyl ether-based structures and new methods for their construction [38–49]. Examination of the synthetic approaches to a wide variety of vinyl ethers has shown serious limitations, such as the use of strong acids or bases, toxic metals, and high temperatures. Therefore, the need to implement new inexpensive and easy methodologies to produce functional vinyl ethers remains attractive. In this context, we expected that the substitution of the bromine atom in molecule **2** by aromatic alcohols as nucleophilic reagents might lead to the desired aryl vinyl ethers **3**. Hence, condensation of allyl bromide **2** with phenol (1.0 equiv.) in the presence of potassium carbonate (1.2 equiv.) and in refluxing acetonitrile afforded the corresponding aryl vinyl ether **3a** in 92% yield after 15 min. In order to generalize the reaction of allyl bromide **2** with various aromatic alcohols, we examined a variety of electronically and sterically substituted phenols. As shown in Table 1, the reaction proceeded smoothly regardless of the steric and electronic properties of these different phenols. With few exceptions, the reaction was generally carried out in 15 minutes in good to excellent yields (Scheme 2, Table 1).

**Scheme 3:**
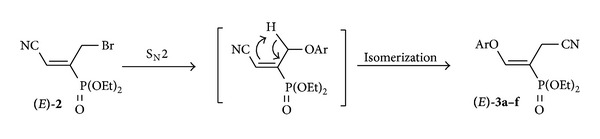
Isomerization of allyl ethers to the corresponding vinyl ones **3**.

The formation of vinyl ethers **3a–f** is the result of the S_N_2 substitution of allyl bromide **2** by different aryloxy groups followed by isomerization. A plausible reaction pathway for the synthesis of these new trisubstituted alkenes **3a–f** is shown in Scheme 3.

The structures of **3a–f** were established on the basis of their ^1^H and ^13^C NMR spectra and by heteronuclear multiple bond correlation (HMBC). Their stereochemistry has been assigned on the basis of NOESY experiment. A sample of **3a** shows no correlation between the vinylic proton (*δ*: 7.53 ppm) and those of CH_2_CN (*δ*: 3.34 ppm). This result indicates that the vinylic proton and the CH_2_ group are on opposite sides of the double bond and therefore the alkene in **3a** is *E* configuration.

**Scheme 4:**
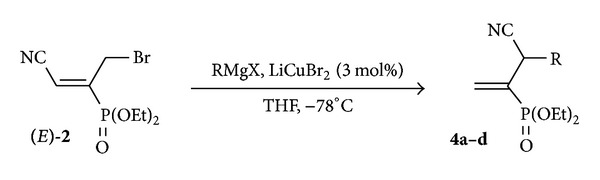
Conjugate addition of Gilman regents on allyl bromide **2**.

This result allowed the consideration of other experimental goals to exploit the electrophilicity of allyl bromide **2** by examining its reactivity with other nucleophilic reagents such as organocuprates. Gilman reagents have been widely used for the construction of organic molecules [50–53], since their discovery in 1900. They display an excellent reactivity towards a wide range of electrophiles and readily undergo transmetallation to provide a variety of organometallic species as organocopper derivatives which react well with soft electrophiles and display excellent chemoselectivity [54]. As a follow-up of our research in the direct substitution of functionalized allyl bromide by organocuprates reagents [55], diethyl (*E*)-1-(bromomethyl)-2-cyanovinylphosphonate **2** constituted the ideal intermediate for the synthesis of new 2-cyanoethylphosphonates **4**. As shown in Scheme 4, conjugate addition of dialkyl organocuprates, generated *in situ* at low temperature from Grignard reagents in the presence of LiCuBr_2_, leads to the corresponding Michael acceptors **4** in an S_N_2′ substitution process. The new vinylphosphonates** 4** obtained are summarized in Table 2.

## 3. Conclusion

In summary, a practical and efficient synthesis of new substituted aryl vinyl ethers **3** and diethyl 1-[(alkyl)(cyano)methyl]vinylphosphonates **4** has been developed. The application of diethyl (*E*)-1-(bromomethyl)-2-cyanovinylphosphonate **2** in other processes will be communicated in due course.

## 4. Experimental Section

### 4.1. Materials and Instrumentation

Starting materials and solvents were purchased and used without further purification. ^1^H-NMR and ^13^C-NMR spectra were recorded on a Bruker AMX 300 spectrometer working at 300 MHz, 121 MHz, and 75 MHz, respectively, for ^1^H, ^31^P, and ^13^C with CDCl_3_ as the solvent and TMS as the internal standard. The chemical shifts (**δ**) and coupling constants (*J*) are, respectively, expressed in parts per million (ppm) and hertz (Hz). All NMR spectra were acquired at 25°C. Assignments of proton (^1^H-NMR) and carbon (^13^C-NMR) signals were secured by DEPT 135 and HMBC experiments. Multiplicity of peaks is indicated by the following: s, singlet; d, doublet; dd, doublet of doublets; t, triplet; dt, doublet of triplets; q, quartet; dq, doublet of quartets; hept, heptuplet; m, multiplet. All reactions were monitored by TLC on silica gel plates (Fluka Kieselgel 60 F254, Merck) eluting with the solvents indicated and visualized by a 254 nm UV lamp and aqueous potassium permanganate solution. For column chromatography, Fluka Kieselgel 70–230 mesh was used. The compounds were examined by gas chromatography-mass spectrometry (GC/MS) and spectra were recorded on an Agilent Technologies 6890 N instrument with an Agilent 5973 N mass detector (EI) and an HP5-MS 30 m × 0.25 mm capillary apolar column (stationary phase: 5% diphenyldimethylpolysiloxane film, 0.25 *μ*m). rt indicates the retention time. IR spectra were recorded on a Bruker Vertex 70 FT-IR spectrophotometer. The elementary analyses (C, H, and N) were performed on a Perkin-Elmer Series II CHNS/O Analyzer 2400.

### 4.2. General Procedure for the Synthesis of Aryl Vinyl Ethers (**3a–f**)

A mixture of allyl bromide (***E***)-**1** (282 mg, 1.0 mmol), phenol (1.0 mmol), and powdered anhydrous K_2_CO_3_ (165 mg, 1.2 mmol) in acetonitrile (3 mL) was refluxed for 15 to 45 minutes under a nitrogen atmosphere with stirring. The reaction mixture was allowed to cool and evaporated to dryness, and the residue was extracted with ethyl acetate (3 × 20 mL) and then the organic layer was washed with brine (20 mL), dried over MgSO_4_, and concentrated under reduced pressure. The obtained liquid was purified by column chromatography (Hexane-AcOEt, 1 : 1).

#### 4.2.1. Diethyl (*E*)-[1-Cyanomethyl-2-(phenoxy)]vinylphosphonate (**3a**)

Yellow liquid. Yield: 92%, IR (neat): 3011, 2225, 1713, 1455, 1242 cm^−1^. ^1^H-NMR (300 MHz, CDCl_3_): 7.53 (d, 1H, ^*3*^ *J*_*HP*_ = 12 Hz, =CH); 7.38 (t, 2H, *J* = 9 Hz, aromatic H); 7.20 (t, 1H, *J* = 9 Hz, aromatic H); 7.09 (d, 2H, *J* = 9 Hz, aromatic H); 4.15 (dq, *J* = 7.5 Hz, *J* = 7.5 Hz, 4H, 2OCH_2_); 3.34 (d, 2H, ^*3*^ *J*_*HP*_ = 15 Hz, CH_2_); 1.38 (t, 6H, *J* = 7.5 Hz, 2CH_3_); ^13^C-NMR (75 MHz, CDCl_3_): 157.0 (d, =CH, ^*2*^ *J*_*CP*_ = 27.75 Hz); 156.1 (aromatic C); 130.0 (aromatic CH); 125.2 (aromatic CH); 117.6 (aromatic CH); 116.6 (d, CN, ^*3*^ *J*_*CP*_ = 3.75 Hz); 98.4 (d, =C, ^*1*^ *J*_*CP*_ = 201 Hz); 62.3 (d, 2OCH_2_, ^*2*^ *J*_*CP*_ = 5.25 Hz); 16.2 (d, 2CH_3_, ^*3*^ *J*_*CP*_ = 6.75 Hz); 12.9 (d, CH_2_, ^*2*^ *J*_*CP*_ = 6 Hz); ^31^P-NMR (121 MHz, CDCl_3_): 17.70; GC/MS (EI): rt = 32.21 min, *m/z* = 295 (M+, 54), 239 (58), 94 (100), 77 (78), 65 (55) 51 (36). Anal. calcd for C_14_H_18_NO_4_P (295,10): C, 56.95; H, 6.14; N, 4.74. Found: C, 56.67; H, 5.04; N, 4.03.

#### 4.2.2. Diethyl (*E*)-[1-Cyanomethyl-2-(4-methylphenoxy)]vinylphosphonate (**3b**)

Yellow liquid. Yield: 68%, IR (neat): 3015, 2222, 1715, 1456, 1245 cm^−1^. ^1^H-NMR (300 MHz, CDCl_3_): 7.49 (d, 1H, ^*3*^ *J*_*HP*_ = 12 Hz, =CH); 7.15 (d, 2H, *J* = 9 Hz, aromatic H); 6.98 (d, 2H, *J *= 9 Hz, aromatic H); 4.15 (dq, 4H, *J* = 7.5 Hz, 4H, *J* = 7.5 Hz, 2OCH_2_); 3.33 (d, 2H, ^*3*^ *J*_*HP*_ = 15 Hz, CH_2_); 2.33 (s, 3H, CH_3_); 1.38 (t, 6H, *J* = 7.5 Hz, 2CH_3_); ^13^C-NMR (75 MHz, CDCl_3_): 157.5 (d, =CH, ^*2*^ *J*_*CP*_ = 27.75 Hz); 154.1 (aromatic C); 134.9 (aromatic C); 130.4 (aromatic CH); 117.2 (aromatic CH); 116.7 (d, CN, ^*3*^ *J*_*CP*_ = 3.75 Hz); 97.8 (d, =C, ^*1*^ *J*_*CP*_ = 201 Hz); 62.8 (d, 2OCH_2_, ^*2*^ *J*_*CP*_ = 6 Hz); 20.7 (s, CH_3_); 16.3 (d, 2CH_3_, ^*3*^ *J*_*CP*_ = 6 Hz); 12.9 (d, CH_2_, ^*2*^ *J*_*CP*_ = 6 Hz); ^31^P-NMR (121 MHz, CDCl_3_): 17.91; GC/MS (EI): rt = 37.9 min, *m/z* = 309 (M+, 40), 202 (36), 146 (33), 108 (100), 91 (44), 77 (33), 65 (38). Anal. calcd for C_15_H_20_NO_4_P (309,11): C, 58.25; H, 6.52; N, 4.53. Found: C, 57.98; H, 5.62; N, 4.47.

#### 4.2.3. Diethyl (*E*)-[1-Cyano-2-(3-hydroxyphenoxy)]vinylphosphonate (**3c**)

Yellow liquid. Yield: 84%, IR (neat): 3356, 3013, 2222, 1718, 1454, 1243 cm^−1^. ^1^H-NMR (300 MHz, CDCl_3_): 8.94 (br, s, 1H, OH); 7.62 (d, 1H, ^*3*^ *J*_*HP*_ = 12 Hz, =CH); 7.13 (t, 1H, *J* = 7.5 Hz, aromatic H); 6.72 (s, 1H, aromatic H); 6.70 (d, 1H, *J* = 9 Hz, aromatic H); 6.56 (d, 1H, *J* = 9 Hz, aromatic H); 4.16 (dq, 4H, *J* = 7.5 Hz, *J* = 7.5 Hz, 2OCH_2_); 3.33 (d, 2H, ^*3*^ *J*_*HP*_ = 15 Hz, CH_2_); 1.39 (t, 6H, *J* = 7.5 Hz, 2CH_3_); ^13^C-NMR (75 MHz, CDCl_3_): 158.5 (aromatic C); 157.7 (d, =CH, ^*2*^ *J*_*CP*_ = 29.25 Hz); 156.9 (aromatic C); 130.4 (aromatic CH); 116.6 (d, CN, ^*3*^ *J*_*CP*_ = 3.75 Hz); 112.3 (aromatic CH); 108.6 (aromatic CH); 104.4 (aromatic CH); 96.8 (d, =C, ^*1*^ *J*_*CP*_ = 203.25 Hz); 62.8 (d, 2OCH_2_, ^*2*^ *J*_*CP*_ = 5.25 Hz); 16.2 (d, 2CH_3_, ^*3*^ *J*_*CP*_ = 6.75 Hz); 12.8 (CH_2_, ^*2*^ *J*_*CP*_ = 6 Hz); ^31^P-NMR (121 MHz, CDCl_3_): 18.83; GC/MS (EI): rt = 38.93 min, *m/z* = 311 (M+, 52), 146 (30), 110 (100), 81 (35), 65 (36). Anal. calcd for C_14_H_18_NO_5_P (311,09): C, 54.02; H, 5.83; N, 4.50. Found: C, 53.81; H, 5.75; N, 4.45.

#### 4.2.4. Diethyl (*E*)-[1-Cyanomethyl-2-(4-nitrophenoxy)]vinylphosphonate (**3d**)

Orange liquid. Yield: 88%, IR (neat): 3012, 2220, 1721, 1538, 1456, 1235 cm^−1^. ^1^H-NMR (300 MHz, CDCl_3_): 8.29 (d, 2H, *J* = 9 Hz, aromatic H); 7.61 (d, 1H, ^*3*^ *J*_*HP*_ = 12 Hz, =CH); 7.28 (d, 2H, *J* = 9 Hz, aromatic H); 4.19 (dq, 4H, *J* = 7.5 Hz, 4H, *J* = 7.5 Hz, 2OCH_2_); 3.39 (d, 2H, ^*3*^ *J*_*HP*_ = 15 Hz, CH_2_); 1.40 (t, 6H, *J* = 7.5 Hz, 2CH_3_); ^13^C-NMR (75 MHz, CDCl_3_): 159.8 (aromatic C); 154.2 (d, =CH, ^*2*^ *J*_*CP*_ = 27.75 Hz); 144.7 (aromatic C); 126.1 (aromatic CH); 117.5 (aromatic CH); 116.1 (d, CN, ^*3*^ *J*_*CP*_ = 3.75 Hz); 102.2 (d, =C, ^*1*^ *J*_*CP*_ = 198 Hz); 62.8 (d, 2OCH_2_, ^*2*^ *J*_*CP*_ = 6 Hz); 16.3 (d, 2CH_3_, ^*3*^ *J*_*CP*_ = 6.75 Hz); 13.1 (d, CH_2_, ^*2*^ *J*_*CP*_ = 6 Hz); ^31^P-NMR (121 MHz, CDCl_3_): 16.17; GC/MS (EI): rt = 44.43 min, *m/z* = 340 (M+, 38), 295 (50), 267 (100), 146 (29), 109 (27), 81 (44), 66 (34). Anal. calcd for C_14_H_17_N_2_O_6_P (340,08): C, 49.42; H, 5.04; N, 8.23. Found: C, 49.20; H, 4.97; N, 8.13.

#### 4.2.5. Diethyl (*E*)-[1-Cyanomethyl-2-(3-methoxyphenoxy)]vinylphosphonate (**3e**)

Yellow liquid. Yield: 78%, IR (neat): 3012, 2220, 1720, 1452, 1248, 1037 cm^−1^. ^1^H-NMR (300 MHz, CDCl_3_): 7.52 (d, 1H, ^*3*^ *J*_*HP*_ = 12 Hz, =CH); 7.26 (t, 1H, *J* = 7.5 Hz, aromatic H); 6.74 (d, 1H, *J* = 9 Hz, aromatic H); 6.68 (d, 1H, *J* = 9 Hz, aromatic H); 6.64 (s, 1H, aromatic H); 4.16 (dq, 4H, *J* = 7.5 Hz, *J* = 7.5 Hz, 2OCH_2_); 3.81 (s, 3H, OCH_3_); 3.33 (d, 2H, ^3^ *J*_*HP*_ = 15 Hz, CH_2_); 1.39 (t, 6H, *J* = 7.5 Hz, 2CH_3_); ^13^C-NMR (75 MHz, CDCl_3_): 161.0 (aromatic C); 157.0 (aromatic C); 156.9 (d, =CH, ^*2*^ *J*_*CP*_ = 27.75 Hz); 130.5 (aromatic CH); 116.6 (d, CN, ^*3*^ *J*_*CP*_ = 3.75 Hz); 111.0 (aromatic CH); 109.3 (aromatic CH); 103.7 (aromatic CH); 98.4 (d, =C, ^*1*^ *J*_*CP*_ = 200.25 Hz); 62.3 (d, 2OCH_2_, ^*2*^ *J*_*CP*_ = 5.25 Hz); 55.5 (OCH_3_); 16.2 (d, 2CH_3_, ^*3*^ *J*_*CP*_ = 6 Hz); 12.9 (d, CH_2_, ^*2*^ *J*_*CP*_ = 6 Hz); ^31^P-NMR (121 MHz, CDCl_3_): 17.64. Anal. calcd for C_15_H_20_NO_5_P (325,11): C, 55.38; H, 6.20; N, 4.31. Found: C, 55.14; H, 6.14; N, 4.35.

#### 4.2.6. Diethyl (*E*)-[1-Cyanomethyl-2-(2,4,6-tribromophenoxy)]vinylphosphonate (**3f**)

White solid. Yield: 57%, IR (neat): 3010, 2220, 1712, 1455, 1241 cm^−1^. ^1^H-NMR (300 MHz, CDCl_3_): 7.73 (s, 2H, aromatic H); 7.01 (d, 1H, ^*3*^ *J*_*HP*_ = 12 Hz, =CH); 4.14 (dq, 4H, *J* = 7.5 Hz, 4H, *J* = 7.5 Hz, 2OCH_2_); 3.40 (d, 2H, ^*3*^ *J*_*HP*_ = 15 Hz, CH_2_); 1.39 (t, 6H, *J* = 7.5 Hz, 2CH_3_); ^13^C-NMR (75 MHz, CDCl_3_): 157.5 (d, =CH, ^*2*^ *J*_*CP*_ = 28.5 Hz); 149.9 (aromatic C); 135.3 (aromatic CH); 120.3 (aromatic C); 117.7 (aromatic C); 116.1 (d, CN, ^*3*^ *J*_*CP*_ = 3.75 Hz); 100.0 (d, =C, ^*1*^ *J*_*CP*_ = 198 Hz); 62.5 (d, 2OCH_2_, ^*2*^ *J*_*CP*_ = 5.25 Hz); 16.2 (d, 2CH_3_, ^*3*^ *J*_*CP*_ = 7.5 Hz); 12.9 (d, CH_2_, ^*2*^ *J*_*CP*_ = 5.25 Hz); ^31^P-NMR (121 MHz, CDCl_3_): 16.35; GC/MS (EI): rt = 43.34 min, *m/z* = 531 (M+, 8), 452 (44), 396 (100), 369 (38), 330 (22), 146 (52), 81 (72), 66 (42). Anal. calcd for C_14_H_15_Br_3_NO_4_P (528,83): C, 31.61; H, 2.84; N, 2.63. Found: C, 31.57; H, 2.72; N, 2.59.

### 4.3. Representative Synthetic Procedure of Diethyl 1-[(Alkyl)(cyano)methyl]vinylphosphonates (**4a–d**)

To a mixture of diethyl (*E*)-1-bromomethyl-2-cyanovinylphsphonate **1** (1.25 g, 5 mmol) and 1 M solution of LiCuBr_2_ (0.15 mL, 3 mol %) in dry THF (20 mL) was added dropwise a solution of alkylmagnesium halide (RMgX) at (−78°C) under a nitrogen atmosphere. The resulting mixture was stirred for a few minutes and then quenched with saturated NH_4_Cl solution (10 mL) and extracted with ether (3 × 20 mL). The combined organic layers were dried over MgSO_4_, filtered, and evaporated under reduced pressure. The crude product was purified by flash chromatography on silica gel (Et_2_O/hexane, 9 : 1) to afford diethyl 1-[(alkyl)(cyano) methyl]vinylphosphonates **4a–d**.

#### 4.3.1. Diethyl 3-Cyanopent-1-en-2-ylphosphonate (**4a**)

Yellow Liquid. Yield: 74%. IR (neat): 2226, 1689, 1242 cm^−1^. ^1^H-NMR (300 MHz, CDCl_3_): 6.30 (d, 1H, ^*3*^ *J*_*HP*_ = 21 Hz, =CH); 6.22 (d, 1H, ^*3*^ *J*_*HP*_ = 45 Hz, =CH); 4.12 (dq, 4H, *J* = 7.5 Hz, *J* = 7.5 Hz, 2OCH_2_); 3.56 (dt, 1H, ^*3*^ *J*_*HP*_ = 9 Hz, *J* = 6 Hz, CH); 2.00–1.77 (m, 2H, CH_2_); 1.35 (2t, 6H, *J* = 7.5 Hz, *J* = 7.5 Hz, 2CH_3_); 1.09 (t, 3H, *J* = 7.5 Hz, CH_3_); ^13^C-NMR (75 MHz, CDCl_3_): 137.1 (d, =C, ^*1*^ *J*_*CP*_ = 181.3 Hz); 132.1 (d, =CH_2_, ^*2*^ *J*_*CP*_ = 8.25 Hz); 116.9 (d, CN, ^*3*^ *J*_*CP*_ = 14.25 Hz); 62.4 (d, 2OCH_2_, ^*2*^ *J*_*CP*_ = 6 Hz); 35.9 (d, CH, ^*2*^ *J*_*CP*_ = 16.5 Hz); 25.9 (d, CH_2_, ^*3*^ *J*_*CP*_ = 2.25 Hz); 16.1 (d, 2CH_3_, ^*3*^ *J*_*CP*_ = 6 Hz); 10.9 (CH_3_); ^31^P-NMR (121 MHz, CDCl_3_): 15.54; GC/MS (EI): rt = 42.81 min, *m/z* = 231 (M+, 3), 167 (29), 149 (100), 71 (19), 57 (34). Anal. calcd for C_10_H_18_NO_3_P (231,10): C, 51.94; H, 7.85; N, 6.06. Found: C, 51.69; H, 7.83; N, 6.03.

#### 4.3.2. Diethyl 3-Cyano-4-methylpent-1-en-2-ylphosphonate (**4b**)

Yellow Liquid. Yield: 92%. IR (neat): 2224, 1696, 1243 cm^−1^. ^1^H-NMR (300 MHz, CDCl_3_): 6.31 (d, 1H, ^*3*^ *J*_*HP*_ = 21 Hz, =CH); 6.20 (d, 1H, ^*3*^ *J*_*HP*_ = 45 Hz, =CH); 4.12 (dq, 4H, *J* = 7.5 Hz, *J* = 7.5 Hz, 2OCH_2_); 3.57 (dd, 1H, ^3^ *J*_*HP*_ = 12 Hz, *J* = 6 Hz, CH); 2.28 (hept, 1H, *J* = 6 Hz, CH); 1.34 (t, 6H, *J* = 6 Hz, 2CH_3_); 1.14 (d, 3H, *J* = 6 Hz, CH_3_); 0.98 (d, 3H, *J* = 6 Hz, CH_3_); ^13^C-NMR (75 MHz, CDCl_3_): 134.1 (d, =C, ^*1*^ *J*_*CP*_ = 176.25 Hz); 132.9 (d, =CH_2_, ^*2*^ *J*_*CP*_ = 7.5 Hz); 117.7 (d, CN, ^*3*^ *J*_*CP*_ = 15.75 Hz); 62.5 (d, 2OCH_2_, ^*2*^ *J*_*CP*_ = 12.75 Hz); 42.4 (d, CH, ^*2*^ *J*_*CP*_ = 17.25 Hz); 29.4 (^*3*^ *J*_*CP*_ = 1.5 Hz, CH); 21.5 (CH_3_); 16.3 (d, 2CH_3_, ^*3*^ *J*_*CP*_ = 6 Hz); ^31^P-NMR (121 MHz, CDCl_3_): 15.63; GC/MS (EI): rt = 24.16 min, *m/z* = 244 (M-1, 3), 203 (33), 175 (24), 147 (100), 81 (11), 66 (24). Anal. calcd for C_11_H_20_NO_3_P (245,12): C, 53.87; H, 8.22; N, 5.71. Found: C, 53.54; H, 7.48; N, 5.79.

#### 4.3.3. Diethyl 3-Cyano-5-methylhex-1-en-2-ylphosphonate (**4c**)

Colorless liquid. Yield: 88%. IR (neat): 2226, 1693, 1244 cm^−1^. ^1^H-NMR (300 MHz, CDCl_3_): 6.3 (d, 1H, ^*3*^ *J*_*HP*_ = 24 Hz, =CH); 6.23 (dd, 1H, ^*3*^ *J*_*HP*_ = 45 Hz, *J* = 3 Hz, =CH); 4.12 (dq, 4H, *J* = 7.5 Hz, *J* = 7.5 Hz, 2OCH_2_); 3.56 (dt, 1H, ^*3*^ *J*_*HP*_ = 9 Hz, *J* = 6 Hz, CH); 1.60 (hept, 1H, *J* = 6 Hz,CH); 1.40-1.26 (m, 2H, CH_2_); 1.35 (2t, 6H, *J* = 7.5 Hz, *J* = 7.5 Hz, 2CH_3_); 0.92 (2d, 6H, *J* = 6 Hz, 2CH_3_); ^13^C-NMR (75 MHz, CDCl_3_): 134.8 (d, =C, ^*1*^ *J*_*CP*_ = 181.5 Hz); 132.5 (d, =CH_2_, ^*2*^ *J*_*CP*_ = 8.25 Hz); 119.3 (d, CN, ^*3*^ *J*_*CP*_ = 13.5 Hz); 62.5 (d, 2OCH_2_, ^*2*^ *J*_*CP*_ = 8.25 Hz); 35.7 (d, CH_2_, ^*3*^ *J*_*CP*_ = 1.5 Hz, CH_2_); 34.5 (d, CH, ^*2*^ *J*_*CP*_ = 16.5 Hz); 27.5 (CH); 22.5 (CH_3_); 22.1 (CH_3_); 16.2 (d, 2CH_3_, ^*3*^ *J*_*CP*_ = 6.75 Hz); ^31^P-NMR (121 MHz, CDCl_3_): 15.65; GC/MS (EI): rt = 28.04 min, *m/z* = 272 (M-1, 5), 233 (43), 203 (32), 174 (30), 160 (19), 147 (100), 138 (27), 66 (36). Anal. calcd for C_13_H_24_NO_3_P (259,28): C, 55.59; H, 8.55; N, 5.40. Found: C, 56.44; H, 8.43; N, 5.35.

#### 4.3.4. Diethyl 3-Cyano-4-(trimethylsilyl)but-1-en-2-ylphosphonate (**4d**)

Yellow liquid. Yield: 78%. IR (neat): 2223, 1711, 1242 cm^−1^. ^1^H-NMR (300 MHz, CDCl_3_): 6.10 (dd, 1H, ^*3*^ *J*_*HP*_ = 48 Hz, *J* = 3 Hz =CH); 6.08 (d, 1H, ^*3*^ *J*_*HP*_ = 21 Hz, =CH); 3.97 (dq, 4H, *J* = 7.5 Hz, *J* = 7.5 Hz, 2OCH_2_); 3.56 (m, 1H, CH); 1.96-1.8 (2 m, 2H, CH_2_); 1.20 (2t, 6H, *J* = 6 Hz, *J* = 6 Hz, 2CH_3_); 0.00 (s, 9H, SiMe_3_); ^13^C-NMR (75 MHz, CDCl_3_): 139.2 (d, =C, ^*1*^ *J*_*CP*_ = 180 Hz); 132.6 (d, =CH_2_, ^*2*^ *J*_*CP*_ = 8.25 Hz); 121.8 (d, CN, ^*3*^ *J*_*CP*_ = 12 Hz); 62.5 (d, 2OCH_2_, ^*2*^ *J*_*CP*_ = 9 Hz); 29.4 (d, CH, ^*2*^ *J*_*CP*_ = 15.75 Hz); 21.4 (d, CH_2_, ^*3*^ *J*_*CP*_ = 2.25 Hz); 16.2 (d, 2CH_3_, ^*3*^ *J*_*CP*_ = 6.75 Hz); −1.57 (s, SiMe_3_); ^31^P-NMR (121 MHz, CDCl_3_): 15.69. Anal. calcd for C_12_H_24_NO_3_PSi (289,13): C, 49.81; H, 8.36; N, 4.84. Found: C, 49.60; H, 8.07; N, 4.76.

## Figures and Tables

**Table 1:** Synthesis of functional aryl vinyl ethers **3a–f**.

Product	Ar	Time (min)	Yield (%)^a^
**3a**	Phenyl	15	92
**3b**	4-Methylphenyl	15	68
**3c**	3-Hydroxyphenyl	30	84
**3d**	4-Nitrophenyl	15	88
**3e**	3-Methoxyphenyl	45	78
**3f**	2,4,6-Tribromophenyl	15	57

^a^Yields refer to isolated products characterized by ^1^H, ^13^C NMR spectroscopy.

**Table 2:** Synthesis of diethyl 1-[(alkyl)(cyano)methyl]vinylphosphonates **4a–d**.

Product	RMgX (equiv.)	Yield (%)^a^
**4a**	EtMgBr (1.5)	74
**4b**	^*i*^PrMgBr (1.5)	92
**4c**	^*i*^BuMgBr (2)	88
**4d**	Me_3_SiCH_2_MgCl (1.5)	78

^a^Isolated yields obtained after purification by chromatography.
